# Characterizing physician directory data quality: variation by specialty, state, and insurer

**DOI:** 10.1186/s12913-024-11269-5

**Published:** 2024-07-18

**Authors:** Neel M. Butala, Kuldeep Jiwani, Emily M. Bucholz

**Affiliations:** 1 Rocky Mountain Regional VA Medical Center, 1700 N Wheeling St, Aurora, CO 80045 USA; 2 University of Colorado School of Medicine, Aurora, CO USA; 3 Hilabs, Inc, Bethesda, MD USA; 4 Children’s Hospital Colorado, Aurora, CO USA

**Keywords:** Physician directory, Health informatics, Health plan, Information technology infrastructure, Health insurance

## Abstract

**Background:**

As U.S. legislators are urged to combat ghost networks in behavioral health and address the provider data quality issue, it becomes important to better characterize the variation in data quality of provider directories to understand root causes and devise solutions. Therefore, this manuscript examines consistency of address, phone number, and specialty information for physician entries from 5 national health plan provider directories by insurer, physician specialty, and state.

**Methods:**

We included all physicians in the Medicare Provider Enrollment, Chain, and Ownership System (PECOS) found in ≥ 2 health insurer physician directories across 5 large national U.S. health insurers. We examined variation in consistency of address, phone number, and specialty information among physicians by insurer, physician specialty, and state.

**Results:**

Of 634,914 unique physicians in the PECOS database, 449,282 were found in ≥ 2 directories and included in our sample. Across insurers, consistency of address information varied from 16.5 to 27.9%, consistency of phone number information varied from 16.0 to 27.4%, and consistency of specialty information varied from 64.2 to 68.0%. General practice, family medicine, plastic surgery, and dermatology physicians had the highest consistency of addresses (37-42%) and phone numbers (37-43%), whereas anesthesiology, nuclear medicine, radiology, and emergency medicine had the lowest consistency of addresses (11-21%) and phone numbers (9-14%) across health insurer directories. There was marked variation in consistency of address, phone number, and specialty information by state.

**Conclusions:**

In evaluating a large national sample of U.S. physicians, we found minimal variation in provider directory consistency by insurer, suggesting that this is a systemic problem that insurers have not solved, and considerable variation by physician specialty with higher quality data among more patient-facing specialties, suggesting that physicians may respond to incentives to improve data quality. These data highlight the importance of novel policy solutions that leverage technology targeting data quality to centralize provider directories so as not to not reinforce existing data quality issues or policy solutions to create national and state-level standards that target both insurers and physician groups to maximize quality of provider information.

**Supplementary Information:**

The online version contains supplementary material available at 10.1186/s12913-024-11269-5.

## Introduction

Patients rely on health insurer provider directories to find physicians and access the care they need, but these directories have a high rate of inaccuracies [1–5]. Poor quality data in provider directories can lead to difficulties in accessing care since patients may be directed to incorrect phone numbers and addresses. Additionally, health insurer provider directory inaccuracies can lead to surprise bills if patients visit a provider that they believe is covered in their network based on inaccurate directory information, but the provider is not actually covered. More broadly, inaccurate provider directories lead to misrepresentation of network breadth and depth for consumers as they choose health plans.

Inaccurate provider directories can also lead to adverse effects at a health system level. In countries with large private healthcare systems in which many individuals depend on commercial health insurance, governments rely on provider directories to ensure that health insurers have contracted with an adequate number of providers to provide their members the option of receiving comprehensive care [6]. In countries with large public healthcare systems, governments can rely on provider directory information to engage in healthcare workforce planning across multiple health disciplines. Current data on physicians, nurses, dentists, and other health professionals from provider directories with information on the range of services offered are a crucial input to health workforce planning frameworks, regardless of the methodology chosen [7]. As such, high-quality provider directory data are necessary to enhance health workforce planning and make decisions regarding the targeted number and mix of professions and skillsets.

A U.S. recent study found that address and specialty information was inconsistent for over 80% of physicians across directories of 5 large national health insurers [8]. In the U.S., health policy solutions to improve provider directory accuracy have been attempted, but they have largely been unsuccessful in achieving their aim. Most states have laws requiring health plans to keep their directories updated [9]. Additionally, the No Surprises Act, as a part of the 2021 Consolidated Appropriations Act, created specific requirements for health plans regarding accuracy and timely updating of provider directories. However, enforcement of these regulations has been minimal [9].

In the U.S. Congress, there have been renewed calls by legislators to address provider directory data quality, particularly as it relates to ‘ghost networks’ [10]. Notably, the U.S. Senate Finance Committee recently passed the Requiring Enhanced and Accurate Lists of (REAL) Health Providers Act, which aims to ensure that Medicare Advantage plans maintain accurate directories of providers, with bipartisan support [11]. Ghost networks are physicians listed in provider directories but, in reality, are not accepting new patients or are unavailable for other reasons [12]. The presence of ghost physician entries is a byproduct of provider directory inaccuracies and can complicate access to physicians for the most vulnerable patients.

As U.S. legislators are urged to combat ghost networks and address the provider data quality issue, it becomes important to better characterize the variation in consistency of provider directories to understand root causes and inform solutions. The aim of this manuscript was to examine variation in consistency of address, phone number, and specialty information for physician entries from 5 national health plan provider directories by insurer, physician specialty, and state. These results can inform the development of novel federal and state-specific policies and guide insurers’ efforts to update provider directories.

## Methods

### Cohort

We searched the Medicare Provider Enrollment, Chain, and Ownership System (PECOS) database for all physicians who were included in the online provider directories of 5 large national health insurers: UnitedHealth, Elevance (formerly Anthem), Cigna, Aetna, and Humana, based on physician name and zip code in September 2022. Once identified in insurer directories, we used the National Provider Identifier number to distinguish between different physicians with the same name in the same zip code. This study was not considered human subjects research as all data were available in publicly accessible health insurer provider directories and therefore there was no interaction or interventions with any individuals or use of any private information. Therefore, the need for ethics approval and informed consent to participate was waived from Colorado Multiple Institutional Review Board review. All methods were performed in accordance with the relevant guidelines and regulations.

### Variables

Among physicians with address information found in ≥ 2 health insurer directories, we compared consistency of physician practice street address across health insurer directories using an approximate matching algorithm, as previously described [8]. Specifically, this algorithm was validated internally through iterative manual review of 200 entries and externally through contacting a sample of 600 entries directly with correct classification in 99%. In evaluating consistency of physician address information across health insurer directories, we did not penalize for differences in abbreviations or punctuation or evaluate secondary unit identifiers, such as suite number.

Among physicians with phone number information found in ≥ 2 health insurer directories, we evaluated consistency of physician phone number information across health plan directories. All digits of a phone number had to match exactly to be considered consistent.

Among physicians with specialty information found in ≥ 2 health insurer directories, we evaluated consistency of physician specialty information across health plan directories according to the 2021 National Uniform Claim Committee taxonomy, as previously described [8]. We categorized specialty information into 31 classifications according to the 2021 National Uniform Claim Committee taxonomy to account for differences in presence of specialty or subspecialty information across health insurer directories [13].

A physician’s information was deemed to be consistent if it was the same among all locations, phone numbers, or specialties across all health insurer directories in which the physician’s information was found. A physician’s information was considered inconsistent if physician address, phone number, or specialty differed across directories or if a physician was found in a directory but an address, phone number, or specialty present in other directories was missing from that directory.

Consistency of address, phone number, and specialty for each physician was computed independently and were not conditional on consistency of other variables.

### Statistical analysis

For each insurer, we calculated the percentage of physicians in that insurer’s directory with consistent address, phone number, or specialty information when including only physicians found in that specific insurer’s physician directory. In order to evaluate consistency of physician information by physician specialty, we calculated the percentage of physicians with consistent address and phone number information by specialty among physicians with consistent specialty information only.

For each state, we calculated the percentage of physicians with consistent address, phone number, or specialty information. We attributed physicians with addresses in multiple states to the most frequent state (mode state) that appeared across all addresses for that physician. Since the presence of insurers in each state may vary, and the consistency of physician information decreases as a physician is found in more directories, we repeated this state-level analysis stratifying by the number of directories in which a physician appeared to account for potential differential ascertainment of consistency information based on the presence of number of insurers in each state.

In supplemental analysis, we additionally evaluated pair-wise consistency of physician directory address, phone number, and specialty information between each individual insurer and PECOS directly.

All analyses were conducted using Python version 3.4 (Python Software Foundation) and SAS version 9.4 (SAS Institute, Cary, NC).

## Results

**Fig. 1:**
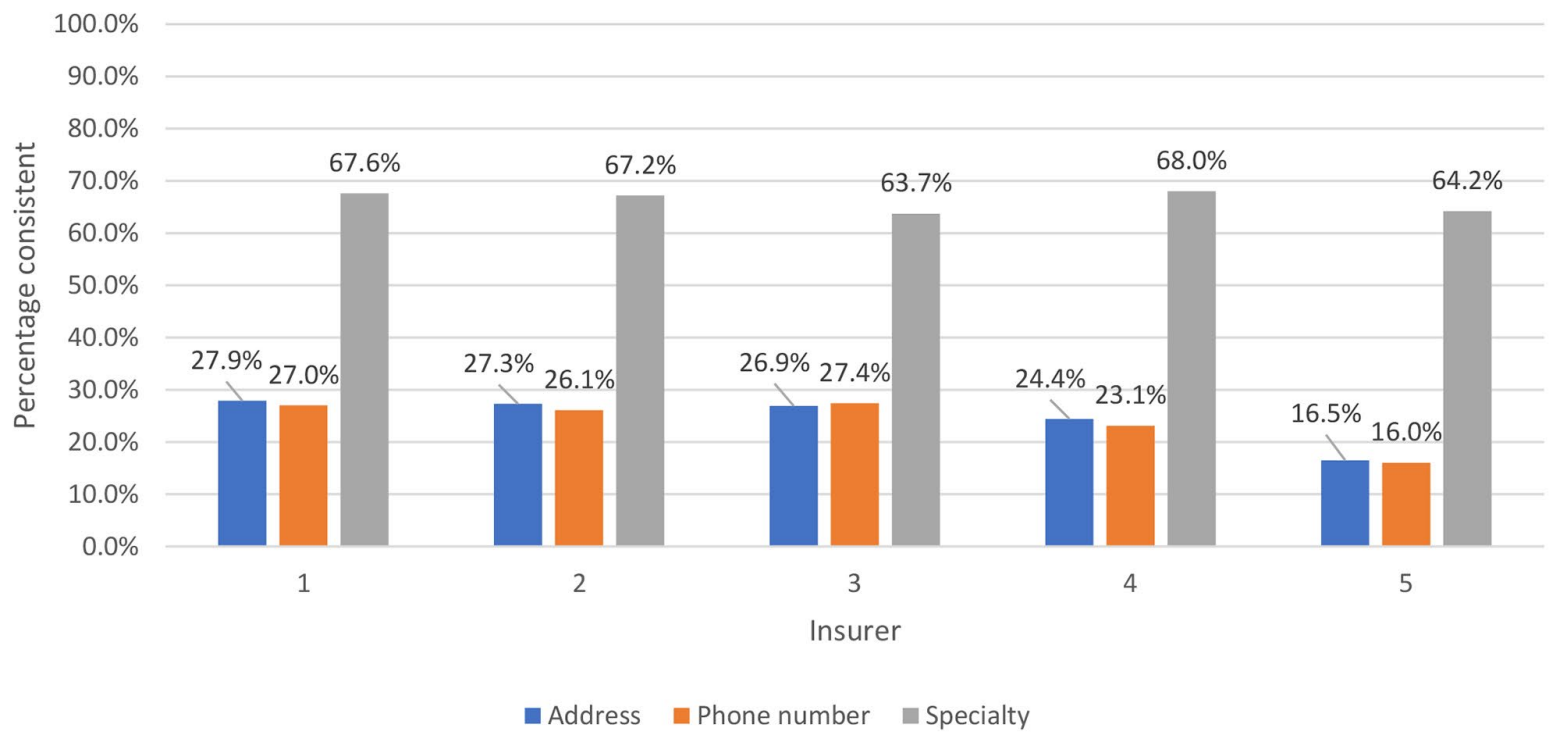
Consistency of physician address, phone number, and specialty information by insurer compared to other insurer directories

Of 634,914 unique physicians in the PECOS database, 449,282 were found in ≥ 2 directories and included in our sample. Consistency of address information varied from 16.5 to 27.9% across insurers, consistency of phone number information varied from 16.0 to 27.4%, and consistency of specialty information varied from 64.2 to 68.0% across insurers (Fig. 1). Similar patterns were observed when each individual insurer was compared to PECOS directly, though address consistency was higher (ranging from 41 to 50% across insurers), phone number consistency was lower (ranging from 14 to 32% across insurers), and specialty consistency was higher (ranging from 80 to 87% across insurers; Supplemental Fig. 1).

Among physicians with consistent specialty information, address and phone number consistency varied considerably by physician specialty (Table 1). General practice, family medicine, plastic surgery, and dermatology physicians had the highest consistency of addresses (37-42%) and phone numbers (37-43%), whereas anesthesiology, nuclear medicine, radiology, and emergency medicine had the lowest consistency of address (11–21%) and phone number (9–14%) information across health insurer directories. Physicians in specialties that deliver primary care (general practice, family medicine, preventive medicine, pediatrics, internal medicine, and obstetrics & gynecology) had consistency of addresses 31% of the time and phone numbers 30% of the time in aggregate.

**Table 1:** Consistency of physician address and phone number information by specialty

Specialty	% address consistent (*n*/*N*)	% phone number consistent (*n*/*N*)
General Practice	41.7% (503/1205)	37% (565/1526)
Family Medicine	41.5% (21,340/51,389)	38.4% (19,688/51,213)
Plastic Surgery	40.7% (624/1535)	41.9% (653/1559)
Dermatology	37% (3346/9050)	40.3% (3639/9028)
Cardiothoracic Surgery	36.3% (225/620)	31% (199/642)
Allergy & Immunology	34.5% (188/545)	36.4% (196/538)
Preventive Medicine	34.3% (23/67)	29.2% (19/65)
Medical Genetics	30.6% (11/36)	39.5% (15/38)
Otolaryngology	29.2% (1749/5985)	32.1% (1916/5978)
Pain Medicine	28.8% (240/833)	29.9% (418/1398)
Pediatrics	28.8% (1229/4270)	24.1% (1020/4235)
Ophthalmology	28.2% (3946/14,014)	31.2% (4366/13,991)
Psychiatry & Neurology	27.7% (3591/12,944)	16.3% (1996/12,273)
Physical Medicine & Rehabilitation	27.4% (1236/4505)	27.2% (1112/4082)
Internal Medicine	26.8% (27,999/104,456)	25.9% (26,679/103,071)
Surgery	26.7% (5015/18,777)	25.8% (4834/18,712)
Pathology	26.5% (445/1677)	17.4% (278/1597)
Neurological Surgery	26.4% (812/3078)	30.7% (943/3070)
Oral & Maxillofacial Surgery	26.2% (292/1113)	19.1% (211/1105)
Obstetrics & Gynecology	25.3% (6404/25,289)	27.2% (6854/25,243)
Orthopedic Surgery	21.7% (3090/14,213)	26.9% (3807/14,165)
Urology	21.1% (1435/6807)	25.3% (1717/6800)
Emergency Medicine	20.8% (478/2303)	14.3% (315/2201)
Anesthesiology	17.4% (927/5317)	14.5% (748/5169)
Nuclear Medicine	14.7% (29/197)	14.3% (111/778)
Radiology	11.7% (1686/14,401)	9.4% (1305/13,852)
**Total**	**28.5% (86,865/304,629)**	**27.7% (83,604/302,329)**

Specialty grouped at “Classification” level the National Uniform Claim Committee Taxonomy. Colorectal surgery and transplant surgery were combined with surgery, hospitalist was combined with internal medicine, and neuromusculoskeletal medicine was combined with psychiatry & neurology. Specialties with < 10 physicians total and physicians with inconsistent specialty information were excluded

**Fig. 2:**
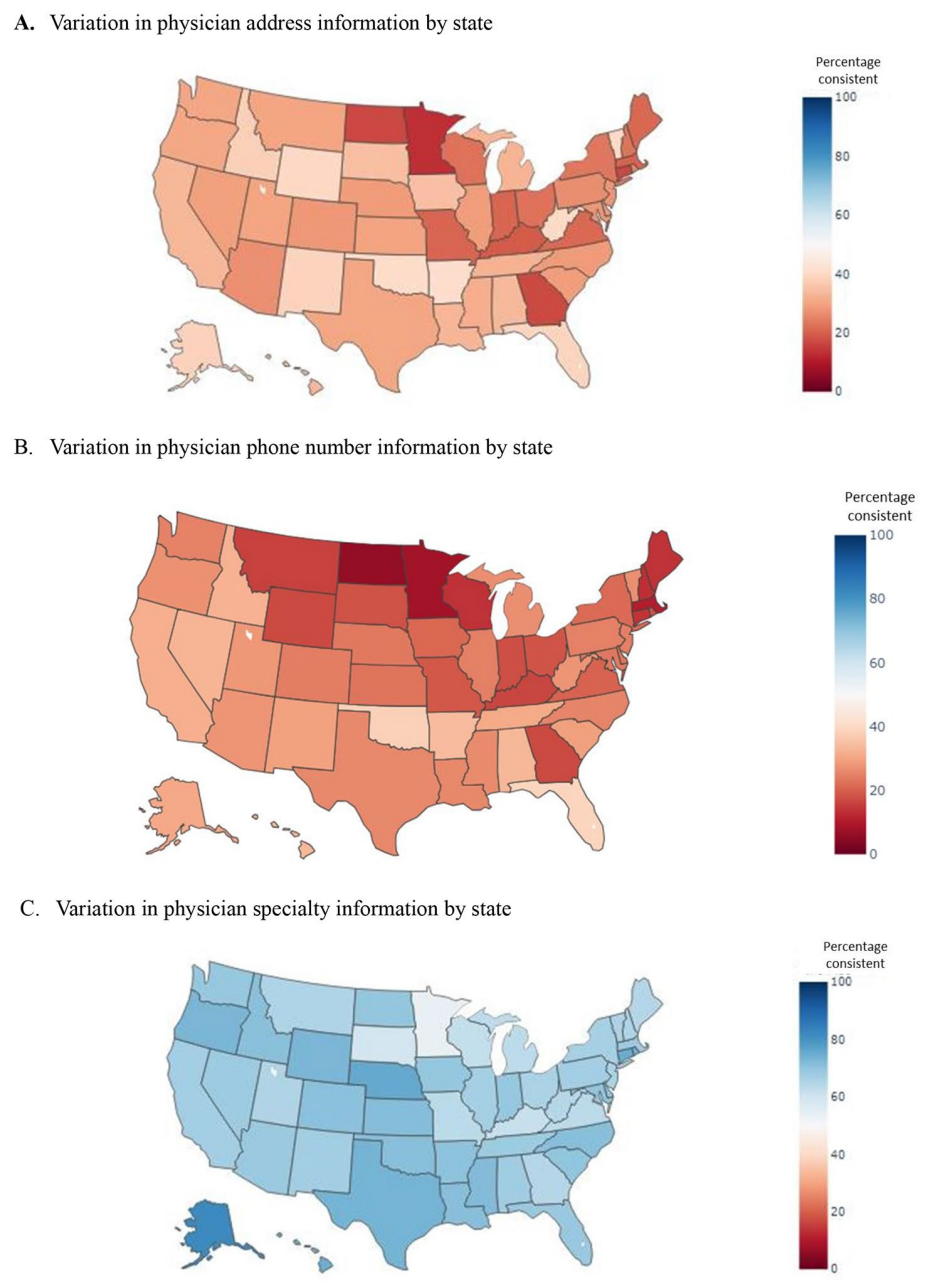
Variation in physician information by state (heat map)

There was marked variation in consistency of address information by state (Fig. 2A), with only 13% of physicians having consistent addresses in Minnesota and 47% of physicians having consistent addresses in Washington, D.C. Similarly, there was marked variation in consistency of phone number information by state (Fig. 2B), with only 6% of physicians having consistent phone numbers in North Dakota and 39% of physicians having consistent phone numbers in Florida. There was less variation in consistency of specialty information by state (Fig. 2C), though this still ranged from 54% of physicians having consistent specialty information in Minnesota to 82% of physicians having consistent specialty information in Alaska. Similar patterns across states were observed when stratifying by number of directories in which a physician was found (Supplemental Figs. 2–4).

## Discussion

In evaluating a large national sample of U.S. physicians, we found considerable variation in consistency of health plan directory information by specialty and state, but less variation by insurer.

The low variation in physician data consistency by insurer is suggestive of the systemic nature of the provider data quality problem across insurers, irrespective of individual insurer processes. All physician directory information originates from physician practices, which face tremendous administrative burden to send physician information to insurers in distinct formats via disparate mechanisms on different schedules [14]. Prior U.S. legislation, as well as the REAL Health Providers Act bill that is currently under consideration, has primarily targeted insurers to maintain accurate directories; however, newer policy solutions may be more successful if they incorporate provider groups as well to address another source of the provider data quality problem.

The magnitude of inconsistency of address and phone number information for physicians in all specialties was high. All specialties had < 50% consistency of physician addresses and phone numbers across health insurer directories examined. Reassuringly, primary care physicians and those that receive many direct patient referrals (plastic surgery and dermatology) had the greatest consistency across addresses and phone numbers, despite often practicing in multiple locations. In contrast, physicians with the lowest consistency across addresses and phone numbers were those that rarely had direct patient referrals (anesthesiology, nuclear medicine, radiology, emergency medicine), for which a health plan provider directory may be less important to ensure access to care. These data suggest that physician practices may be responsive to incentives to improve provider directory accuracy, given that specialties with a higher degree of interface with patients often had better provider data quality. Future policy solutions could leverage physician incentives further to improve directory quality.

We found considerable variation in address, phone number, and specialty data quality by state. However, the key drivers of this variation are unclear. States vary in their enforcement strategy for national laws pertaining to provider directory accuracy that target insurers [15]. Additionally, many states have specific laws on health plan provider directory quality targeting insurers, but they are variably enforced [9]. Notably, California has had several documented enforcements of state laws regarding provider directories in recent years [1]. but it remains near the median nationally in terms of provider data quality. Future research into the drivers of state variation in provider directory quality remains a rich area for further inquiry.

In the United States, the Centers for Medicare and Medicaid Services has proposed the creation of a National Directory of Healthcare Providers and Services, which would be a single, centralized system that would aim to reduce the burden for insurers and physicians while promoting real-time accuracy for patients [16]. This unified solution would engage both insurers and physician groups, though would require a radical shift in the way physician data is transmitted between entities. Notably, the PECOS directory is currently meant to be a national “gold standard” source of provider information for physicians who treat Medicare patients, but repeated Office of Inspector General investigations have found this to have substantial inaccuracies [17]. These findings are consistent with the results from the pairwise comparisons between individual insurers and PECOS in our study, which reaffirm the large magnitude of inconsistencies in this existing U.S. national government-run provider directory. An alternative, less-disruptive policy solution that may be easier to implement would be to create a national standard by which to exchange provider directory information, similar to administrative claims.

Internationally, many government agencies maintain centralized provider directories, in part by necessity as a function of operating public healthcare delivery systems, such as the National Health Service in the United Kingdom. In such contexts, accurate provider directory information is a key input to health workforce planning, which has implications for the short-term allocation of the healthcare workforce as well as the long-term development of the healthcare workforce through targeted investment in training programs. Some evidence suggests that inaccuracies are also present in high magnitude in such government-maintained directories [18, 19], though a thorough evaluation of provider directory accuracy in most countries is lacking. Recognizing the need to streamline and improve provider data, in early 2023, the Australian Digital Health Agency launched Provider Connect Australia (PCA) [20], which is a unified database that streamlines access to provider information for patients and other healthcare entities. Given many parallels between the healthcare systems of Australia and the United States, it will be important to gauge the success of PCA in enrolling provider groups and the accuracy of its information as the world looks to PCA as a model for forward-thinking technology-enabled national provider directory.

## Conclusions

In this report evaluating health plan physician directory consistency for over 40% of all US physicians, we found minimal variation by insurer and considerable variation by physician specialty and state. These data highlight the importance of novel policy solutions to centralize provider directories or create national and state-level standards. Future legislation should engage both insurers and physician groups to maximize quality of provider information.

### Electronic supplementary material

Below is the link to the electronic supplementary material.

Supplementary Material 1

## Data Availability

Deidentified participant data can be made available at time of publication upon reasonable request to researchers for analyses pertaining to physician directory data quality by contacting the corresponding author.

## Abbreviations

PECOS: Provider Enrollment, Chain, and Ownership System
REAL Health Providers Act: Requiring Enhanced and Accurate Lists of Health Providers Act
