# Supplementary material for: Characterizing physician directory data quality: variation by specialty, state, and insurer
Source: BMC Health Serv Res. 2024 Jul 18;24:808. doi: 10.1186/s12913-024-11269-5 (PMC11256460; doi:10.1186/s12913-024-11269-5)

Supplemental Figure 1. Consistency of physician address, phone number, and specialty information by insurer compared to the Medicare Provider Enrollment, Chain, and Ownership System directory


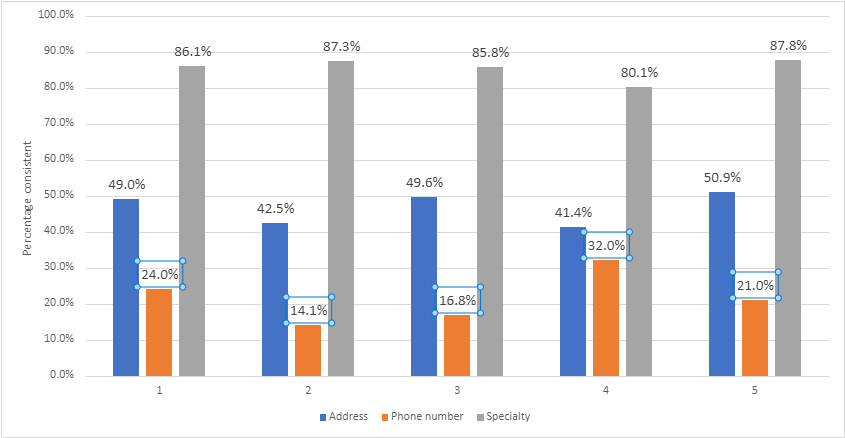


Supplemental Figure 2. Variation in physician address information by state based on number of directories in which physician was found (heat map)

1. Among physicians found in 2 directories


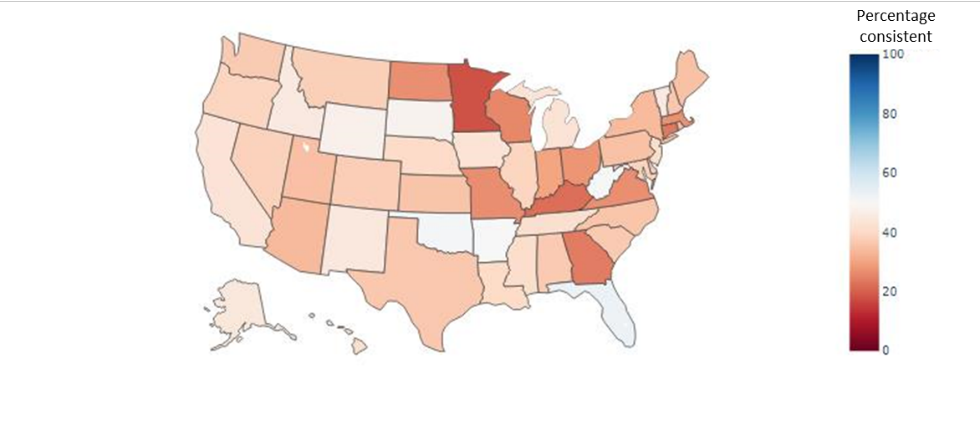


1. Among physicians found in 3 directories


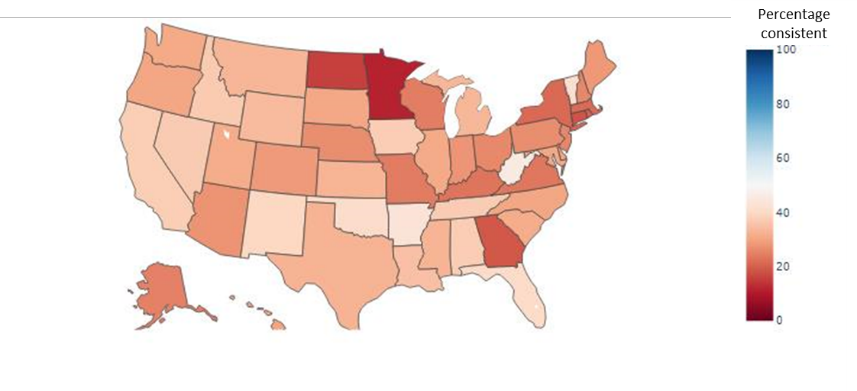


1.
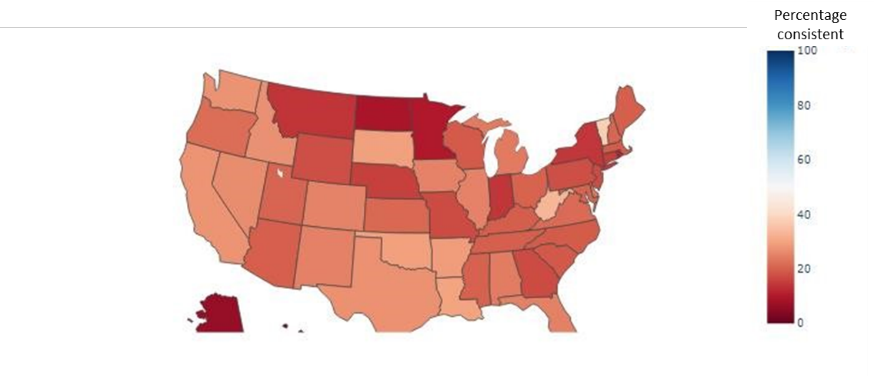
Among physicians found in 4 directories
2.
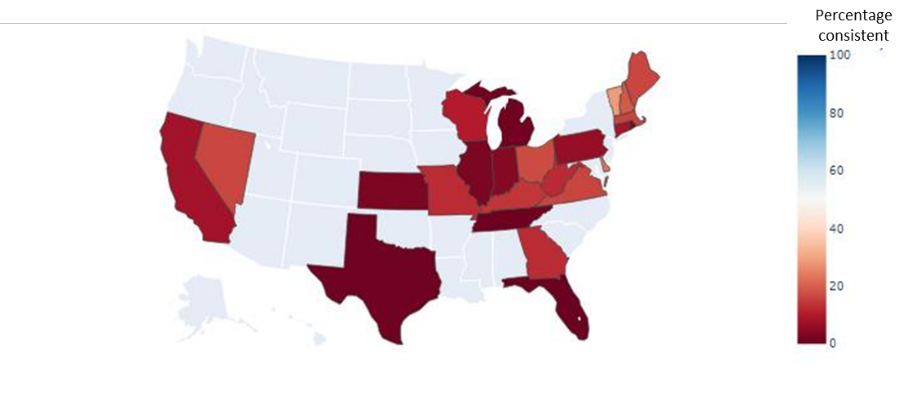
Among physicians found in 5 directories

Supplemental Figure 3. Variation in physician phone number information by state based on number of directories in which physician was found (heat map)

1. Among physicians found in 2 directories


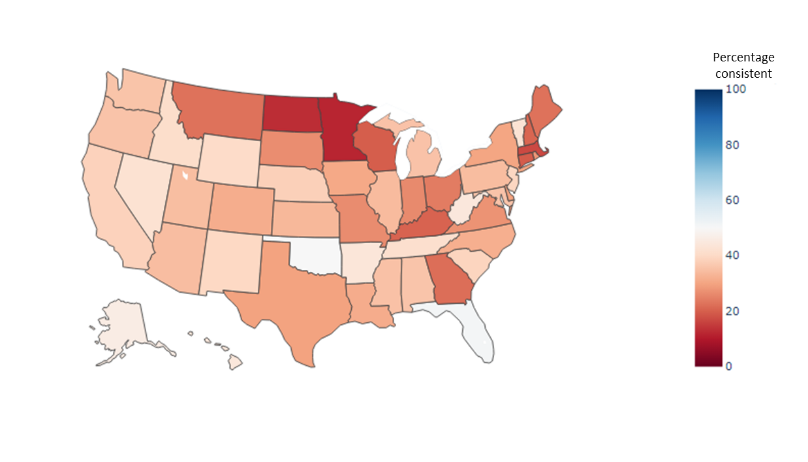


1. Among physicians found in 3 directories


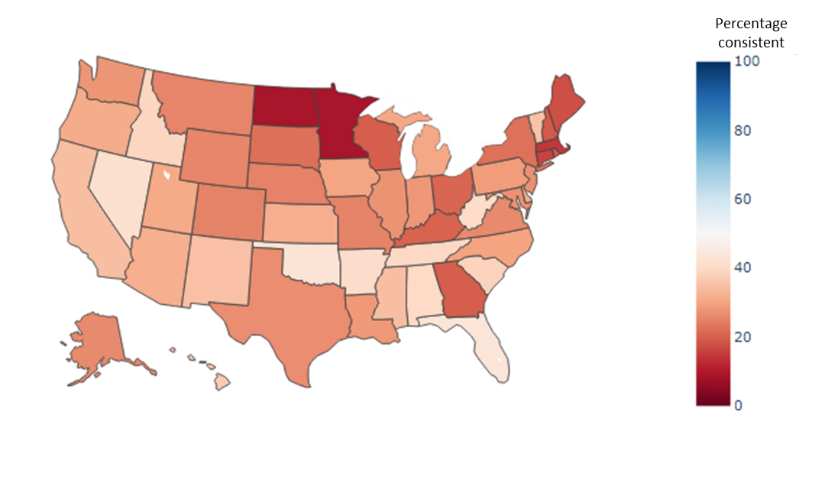


1. Among physicians found in 4 directories


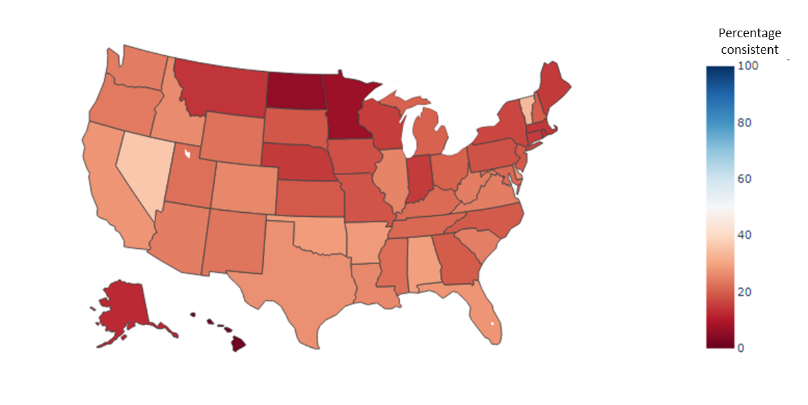


Among physicians found in 5 directories
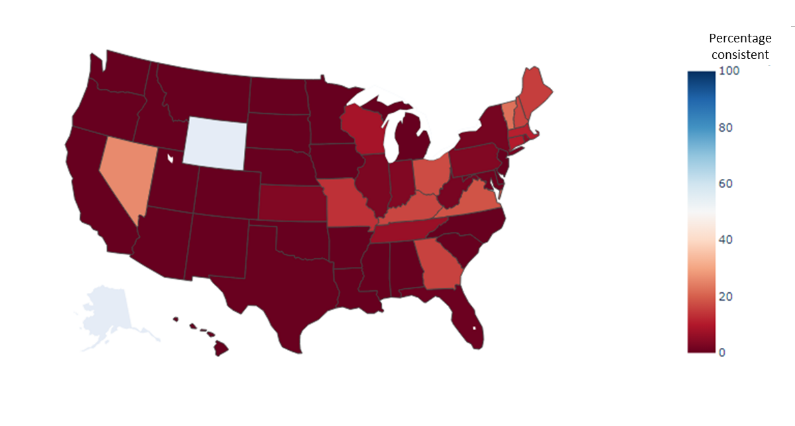


Supplemental Figure 4. Variation in physician specialty information by state based on number of directories in which physician was found (heat map)

1. Among physicians found in 2 directories


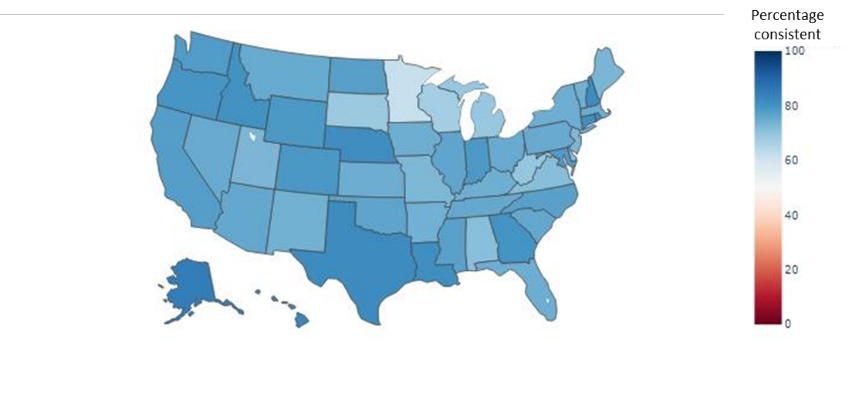


1. Among physicians found in 3 directories


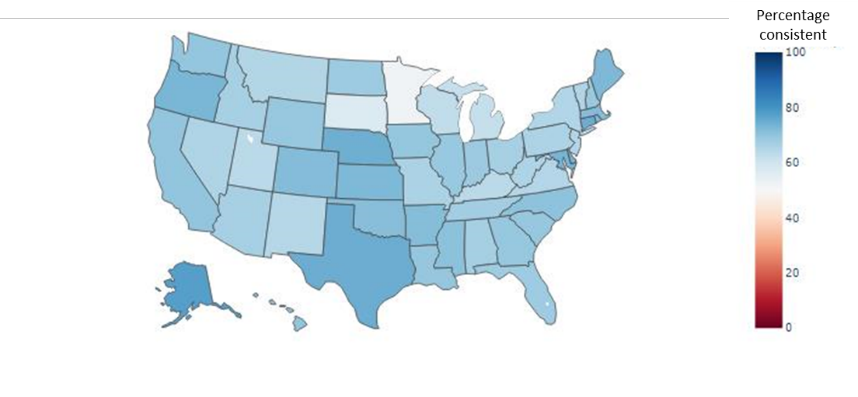


1. Among physicians found in 4 directories


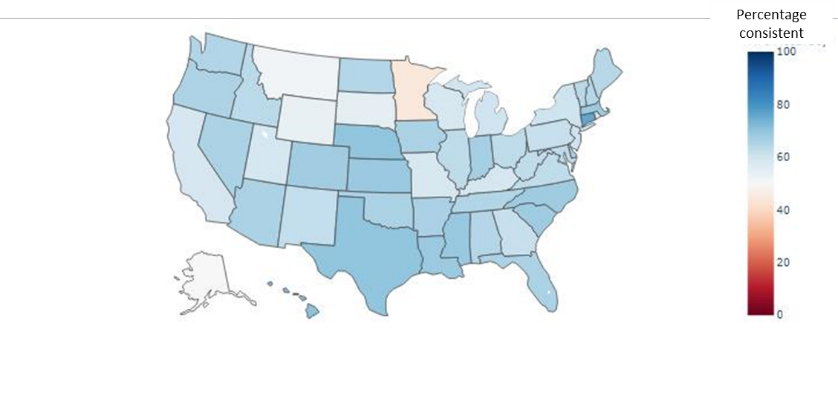


1. Among physicians found in 5 directories


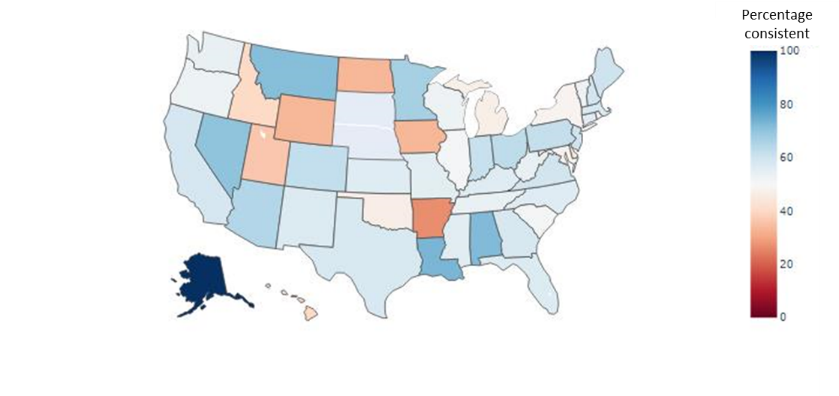

Supplement: Supplementary file 1 — Supplementary Material 1 [file 12913_2024_11269_MOESM1_ESM.docx]
